# Remote coaching for supporting the implementation of treatment for depression in primary care in Madhya Pradesh, India: protocol for a cluster randomized controlled trial

**DOI:** 10.3389/frhs.2024.1477444

**Published:** 2024-09-24

**Authors:** Ameya P. Bondre, Abhishek Singh, Deepak Tugnawat, Dinesh Chandke, Azaz Khan, Ritu Shrivastava, Chunling Lu, Rohit Ramaswamy, Vikram Patel, Anant Bhan, John A. Naslund

**Affiliations:** ^1^ Sangath, Bhopal, India; ^2^ Department of Global Health and Social Medicine, Harvard Medical School, Boston, MA, United States; ^3^ Department of Public Health Leadership and Practice at Gillings School of Global Public Health, University of North Carolina at Chapel Hill, Chapel Hill, NC, United States

**Keywords:** primary care, rural, depression, mental health, implementation, hybrid trial, implementation support strategy

## Abstract

**Background:**

Upwards of ninety percent of individuals living with depression in India do not have access to evidence-based treatments, especially in rural areas. Integrating these treatments into primary care is essential for bridging this care gap. This trial aims to evaluate whether a remote coaching implementation support strategy, referred to as Enhanced Implementation Support, is superior to routine support, referred to as Routine Implementation Support, in supporting the delivery of collaborative depression care in rural primary care centers.

**Methods:**

Employing a cluster-randomized hybrid type-III implementation trial design, 14 primary care facilities in Sehore district, Madhya Pradesh, will implement a collaborative depression care package based on the WHO's mhGAP program. Facilities will be randomized to either Enhanced Implementation Support or the Routine Implementation Support control condition. Enhanced Implementation Support consists of remote coaching and technical assistance, supplemented with in-person visits, and guided by the Plan-Do-Study-Act implementation cycles. The primary implementation outcome is the proportion of outpatients screened for depression by facility staff, with secondary outcomes including the proportions of outpatients who screen positive for depression, are referred to the medical officer, and initiate treatment. Secondary patient outcomes include proportion of patients who achieve reduction in depression symptom severity at 3-month follow up. Acceptability, feasibility, and fidelity of the depression care package will be assessed through routine observations collected during field visits, facility audits, and qualitative exit interviews with facility staff. Costs of delivering the Enhanced Implementation Support strategy will also be estimated.

**Discussion:**

This trial can inform efforts to integrate depression care in rural primary care facilities in a low-resource setting, and illuminate whether external coaching support is superior relative to existing implementation support for achieving these goals.

**Trial Registration:**

NCT05264792.

## Introduction

Mental disorders pose a serious and growing challenge to health systems, with depression representing the leading cause of disability due to mental illness worldwide (1, 2). In India, depression affects over 50 million people, and is correlated with suicide (3) and ischemic heart disease (4). Evidence-based clinical interventions exist for depression (5); however, the gap between those who need and receive treatment, referred to as the care gap (6), is alarming, with upwards of 90% of individuals not having access to care in rural India (7–9).

Integrating evidence-based treatments for depression into primary care represents an essential priority for bridging this care gap in low-income and middle-income countries (LMICs) such as India (10–12). There are multiple barriers to the successful implementation of mental health services into routine care settings in LMICs related to legislation and policy, financing and resources, organization and planning, and ensuring necessary workforce capacity (13–15). In India, government efforts to integrate evidence-based depression care into routine care settings have faced challenges due to suboptimal organization and planning (13), emphasis on top-down “one size fits all” approaches to service delivery, limited attention to collaborative care models, and inadequate training and support for primary care personnel (16, 17). Efforts to overcome these challenges have demonstrated success through the use of lay health counsellors (18) and have resulted in high follow-up rates, and early remission and recovery among patients (14). However, significant limitations persist, including insufficient engagement of community-level health workers, low utilisation of evidence-based psychological interventions, and inability to sustain delivery of these programs due to overreliance on external resources and few available specialized providers (14, 16).

Novel approaches are needed to support frontline health workers within existing government primary health care facilities to ensure uptake and sustained delivery of depression care. Drawing from the implementation science literature, there is mounting evidence showing that “implementation strategies” can facilitate the integration of proven interventions into routine practice (19–22), including in LMICs (22). Implementation strategies refer to techniques, often guided by a theory or framework, to enable the adoption and implementation of an evidence-based clinical intervention in practice (23, 24). While there has been an increasing emphasis on examining the integration, acceptability, feasibility and cost of treating depression in various settings in India (22–25), there remains a paucity of studies employing rigorous randomized controlled designs, and few that have evaluated use of a comprehensive implementation strategy at the primary care level.

This trial seeks to address this knowledge gap through use of a cluster-randomized controlled superiority trial design to evaluate whether a “remote coaching implementation support strategy” is superior when compared to “routine implementation support” in facilitating the delivery of collaborative depression care in primary care facilities. Successful delivery of depression care will be defined by increased rates of screening for depression (i.e., primary implementation outcome) and detection of depression cases and initiation of treatment (i.e., secondary implementation outcomes) in the participating primary care facilities. Costs of the implementation support strategy will also be assessed, as well as secondary patient-level clinical and functional outcomes. This trial builds on recent health system-level changes in India, where the screening and management of non-communicable diseases (NCD) now form part of essential primary care services, yielding an opportunity for integrating depression care (26). This trial will employ routine health facility cadres, such as the auxiliary nurse midwife (ANM) and nurses, primarily for depression screening, and the medical officer (MO) for diagnosis, treatment and referral of cases (either to the District Mental Health Program for specialist-delivered care, or to a brief psychosocial intervention for depression delivered by a trained community health worker, referred to as an Accredited Social Health Activist), and employ routine data collection at the facility-level.

### Study objectives and hypotheses

The objective of this hybrid type III cluster-randomized controlled superiority trial is to evaluate whether a remote coaching implementation support strategy, referred to as “Enhanced Implementation Support”, is superior when compared to routine support, referred to as “Routine Implementation Support”, in facilitating the delivery of depression care in primary care facilities in rural India. This will be ascertained by measuring the proportion of outpatients screened for depression, number of cases of depression detected, and number of patients referred to the MO and initiated on treatment at the participating primary care facilities. It is hypothesized that Enhanced Implementation Support will be superior to Routine Implementation Support in increasing the proportion of outpatients screened for depression using the two-item Patient Health Questionnaire (PHQ-2) (27) by ANMs/nurses. It is also hypothesized that Enhanced Implementation Support will be superior to Routine Implementation Support in increasing the number of cases of depression detected, and number of patients referred to the MO and initiated on treatment.

## Methods

### Trial design

This trial will employ a two-arm hybrid type III cluster-randomized controlled superiority trial design (28). Each cluster, or “Primary Health Center (PHC)”, is the unit of randomization, with equal allocation of clusters between arms.

### Trial setting

Since 2011, Sangath has worked closely with the Ministry of Health and Family Welfare, Government of Madhya Pradesh, resulting in the establishment of a Memorandum of Understanding and a significant track record in the region, which serves as the foundation for this project (14, 29). This trial will be implemented in government-run rural PHCs of Sehore district, Madhya Pradesh. Madhya Pradesh has a population over 72 million, of which nearly 73% live in rural areas (30), and is ranked among the lowest on the Human Development Index (31, 32) compared to other Indian states. Sehore district has a population of 1.31 million (33), with a total of 25 PHCs (34). Each PHC serves about 20,000–30,000 people as per the Indian Public Health Standard Guidelines. PHCs were selected as the setting for this trial given ongoing roll out of the *Ayushman Bharat* program, which will upgrade these facilities to “Health and Wellness Centers” aimed at serving as the national platform for delivery of comprehensive NCD care including mental health screening, diagnosis, treatment and referral (35). PHCs offer outpatient services, typically for 6 h each day with an expected caseload of 40 outpatients. Mental health services are not currently provided through PHCs, and the initiation of mental health services under the *Ayushman Bharat* program has not yet started in the study setting as of the trial launch (29).

### Study procedures

In preparation for this trial, PHCs were identified and recruited, and facility staff were trained in the delivery of the depression care package. The PHCs were then randomized to the Enhanced Implementation Support or Routine Implementation Support strategies. This was followed by a 4-month embedding period from May-August 2022 to allow participating facilities to begin delivering depression care, and to ensure data collection procedures could be tested across both intervention and control facilities. For intervention facilities, the embedding period offered the opportunity to train staff in the Enhanced Implementation Support strategy protocol, and to allow sufficient uptake of the strategy ahead of the trial launch. The embedding period also made it possible to understand potential barriers at the facility or system-level, modify the study procedures, and make revisions as needed to the Enhanced Implementation Support remote coaching protocol. The active intervention phase will then last 12-months, followed by a 6-month period for continued data collection from the PHCs.

### Facility (“cluster”) eligibility and recruitment

Of the total PHCs, 16 have been upgraded to “Health and Wellness Centers”, of which 14 are rural and serve as the setting for this trial. These rural PHCs also have a linked Accredited Social Health Activist (ASHA), a cadre of frontline health worker that serves as the link between the PHC and the community, and who will be available to deliver a brief psychosocial intervention for depression, called the Healthy Activity Program (HAP) (36), following previous district-wide training efforts (29, 37). Through consultation with the Ministry of Health and Family Welfare and officials from the National Health Mission, Government of Madhya Pradesh, and the District Chief Medical Health Officer (CMHO), these 14 facilities were invited and enrolled in the trial. The teams at each PHC were briefed on the study details and trained on the depression care package. The characteristics of each PHC were documented, including: average number of adult outpatient attendees per week; size of catchment area population; staff availability and turnover; number of Sub-Health Centres linked to the PHC; distance to nearest higher-level health facility for referrals; and status of the NCD programming, including format and frequency of data reporting, use of digital applications, and status/plans of integration of mental health services.

The teams at all 14 PHCs were trained on the World Health Organization's (WHO) mhGAP intervention guide (5), previously adapted for Sehore district (14). The MOs were trained through in-person and virtual modes, depending on their availability to attend in-person training, over eight days in the diagnosis, treatment and referral of positive cases of depression, including the option for referring patients to receive HAP delivered by ASHAs (29). The training for ANMs and nurses was conducted separately, as part of a 2-day in person training with instruction on screening of outpatients for depression, and referral and tracking of depression cases. Training for ANMs and nurses emphasized case detection and screening outpatients using the PHQ-2, and entering data into a “Screening Record Register” to track screening rates, refusal, patient willingness to be contacted by the research team (to participate if they screen positive), and referral to the MO and initiation of treatment. The PHQ-2 is a screening questionnaire for depression which comprises two items of the widely used and contextually validated PHQ-9. The PHQ-2 was selected to increase the quality and efficiency of screening conducted by ANMs/Nurses, and its use, instead of the full 9 item version, is supported by prior studies showing that the positive predictive value for the presence of “any depressive disorder” is 75% for a score of ≥3 (38). Further, the health system in India has planned to adopt the PHQ-2 for community-level depression screening (39). Each trial arm will have 7 PHCs, with estimated total staffing of 14 MOs (2 per facility), 14 nurses (2 per facility), and 7 ANMs (1 per facility), with some variation in staffing expected between facilities.

### Participant recruitment

Adult outpatients (age ≥18 years) of any gender who screen positive for depression (PHQ-2 ≥ 3) at participating PHCs will be invited to enroll in this trial. Outpatients will be excluded if they have significant speech, hearing, language or cognitive impairment impacting their ability to provide informed consent and complete study assessments, those needing urgent medical or psychiatric attention, those not planning to stay in the study catchment area for at least three months (for participation in the clinical outcome assessment), or those who do not understand Hindi.

**Figure 1:**
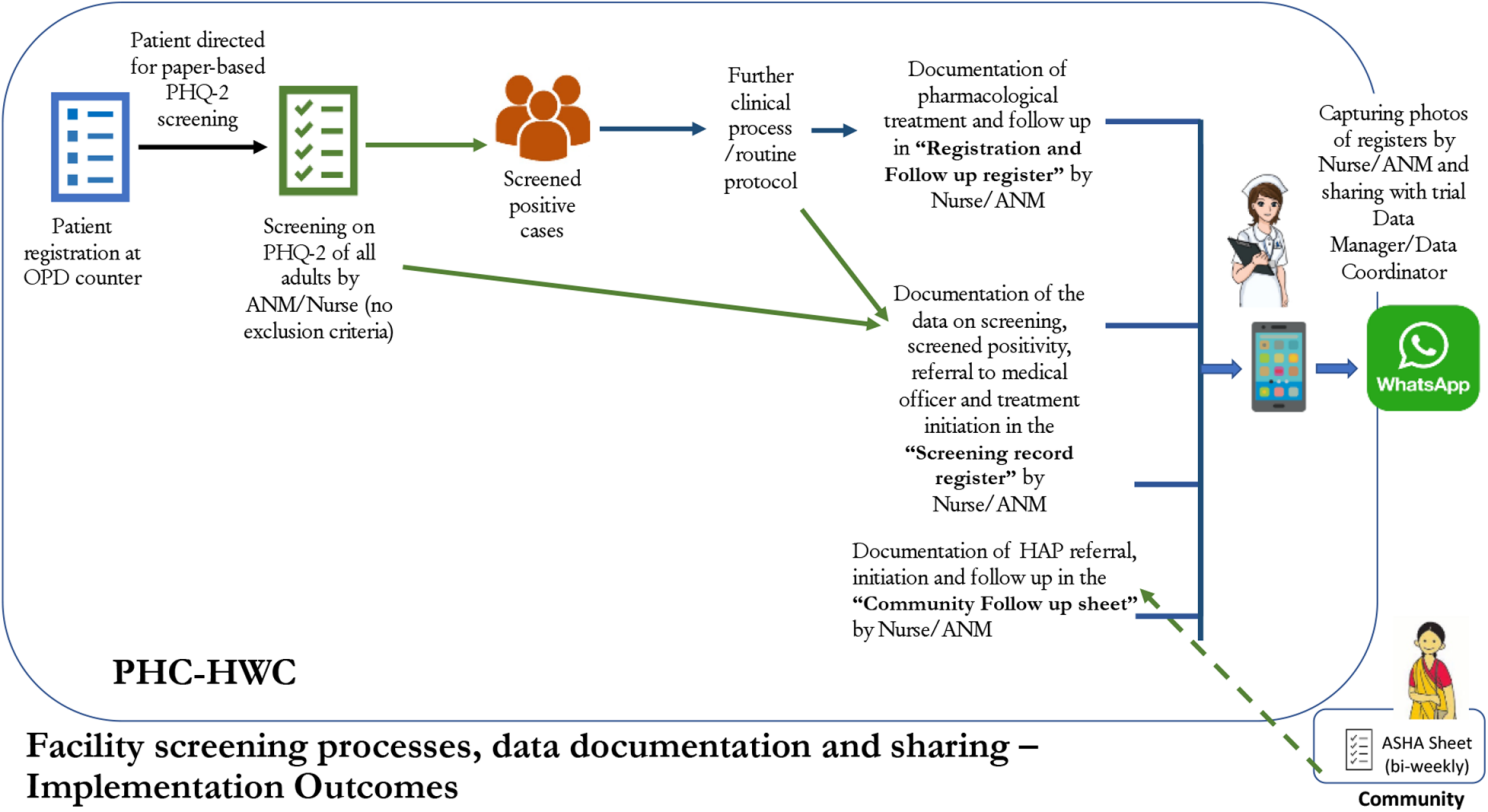
Facility screening processes, data documentation and sharing.

The ANM/nurse will be the first point of contact for determining a patient's willingness to participate in the study. After screening on the PHQ-2, if a patient is screened positive (score ≥3), they are potentially eligible to participate in the clinical care component of the trial. Prior to referral to the MO, the ANM/nurse will mention the study to the patient and ask whether they are willing to be contacted to learn more (Figure 1). If the patient agrees, the ANM/nurse will record their name, address, and contact number to share with the study team. The study data manager will retrieve the data on patient willingness to be contacted during weekly review of facility screening activities, and share this data with a study research assistant. The research assistant will call the patient to introduce the study, and determine whether the patient is interested. If the patient assents, the research assistant will meet the patient within 7 days (up to a maximum of 2 weeks) at the patient's home or a mutually agreed location (e.g., PHC) to confirm eligibility, collect informed consent, and complete baseline assessment.

### Interventions: implementation support strategies

#### Control condition: routine implementation support

All 14 PHCs will receive Routine Implementation Support, referring to the existing implementation strategy utilized by the health system for facilitating the roll out of NCD care services (Table 1). As part of Routine Implementation Support, mental health performance indicators will be integrated into existing NCD monitoring and recorded using a standardized Screening Record Register (Table 2). These indicators will be collated, reviewed and synthesized by the facility teams and the district team as part of existing NCD indicators. The district team is composed of the CMHO, District Program Manager, and the District Community Mobilizer & Monitoring and Evaluation Officer. Each facility shares monthly NCD performance indicators with the district team via email, followed by review and discussion over WhatsApp. The district team also visits the facilities on an as needed basis, and coordinates district-level meetings.

**Table 1:** Routine and enhanced implementation support strategies.

	Routine implementation support	Enhanced implementation support strategy
Who delivers the support?	District program management team	ESSENCE Implementation Support Coaching team
Who is the target of the support?	14 trial facility teams including ANMs and staff Nurses, as well as medical officers in-charge	7 trial facility teams randomized to the intervention arm including ANMs and staff Nurses for improving the screening of depression
Components of support	Mental health data will be reviewed as part of the usual NCD review process. This may include: - Weekly in person facility meetings to review and discuss mental health and other performance indicators - Monthly mental health performance indicator data received by facilities via email - Data submission reminders from District Level team to facilities via common WhatsApp group	Mental health data will be reviewed as part of the usual NCD review process. This may include: - Weekly in person facility meetings to review and discuss mental health and other performance indicators - Monthly mental health performance indicator data received by facilities via email - Data submission reminders from District Level team to facilities via common WhatsApp group In addition, the Enhanced Implementation Support Strategy includes: (1) **Technical assistance** remote coaching sessions delivered by ESSENCE Implementation Coaching Support team to review depression screening performance indicator data with facility team *(every two weeks)* (2) Cross-facility **collaborative virtual learning conferences** moderated by ESSENCE Implementation Support Team *(planned monthly)* (3) Cross-facility WhatsApp group for **remote peer-to-peer collaborative learning** moderated by ESSENCE Implementation Support team *(throughout intervention)*

**Table 2:** Indicators of successful integration of depression care.

List of indicators collected from all facilities
1. Proportion of patients screened on PHQ-2
2. Proportion of patients who refused PHQ-2 screening
3. Proportion of patients screened positive on PHQ-2 (scored ≥3) or “cases”
4. Proportion of cases who agreed to be called by the Research Assistant (“assent”)
5. Proportion of cases referred for MO consultation
6. Proportion of cases who received antidepressant
7. Proportion of cases referred to District Mental Health Program
8. Proportion of cases referred to the ASHA for brief psychosocial intervention (i.e., HAP)
9. Proportion of cases with scheduled followed up for antidepressant medication
10. Proportion of cases who followed up for antidepressant medication
11. Proportion of cases who missed follow up for antidepressant medication
12. Proportion of cases initiated on HAP
13. Proportion of HAP follow-up sessions delivered of those scheduled
14. Proportion of loss to follow-up cases in HAP of those initiated into HAP
15. Proportion of HAP treatments closed of those initiated into HAP
16. Total Adult OPD (outpatient department) attendance, or total number of adult patients attending the outpatient clinic

Data sourced from the registers and received by the Data Coordinator via WhatsApp from all 14 facilities will ensure tracking of these indicators in both arms. These metrics will also be used to guide the coaching sessions for the 7 facilities receiving the Enhanced Implementation Support intervention.

#### Intervention condition: enhanced implementation support

The seven PHCs randomized to the intervention arm will receive the Enhanced Implementation Support strategy consisting of individualized coaching support, in addition to the components of Routine Implementation Support described above. There is one lead coach and two support coaches, who are members of the research team with prior knowledge and experience working with the health system. No additional coaching staff will be involved in the enhanced implementation support activities, and there will be no coaching staff from the health system. Following a train-the-trainer model, the lead coach will receive a 2-day remote training offered by a member of the investigator team with expertise in implementation science. The training will consist of didactic sessions and roleplay exercises using a decision-making flowchart to guide the technical assistance coaching sessions with the facility teams. The lead coach will then train the support coaches in a 2-day in person training covering the same content and activities. The coach trainings will take place ahead of the trial launch. Drawing from the Institute of Healthcare Improvement's (IHI's) Breakthrough Series model, which recommends that collaboratives meet for a 6–15 month period to support organizations in making “breakthrough” improvements in quality (40, 41), a 12-month intervention period was selected for this trial.

**Figure 2:**
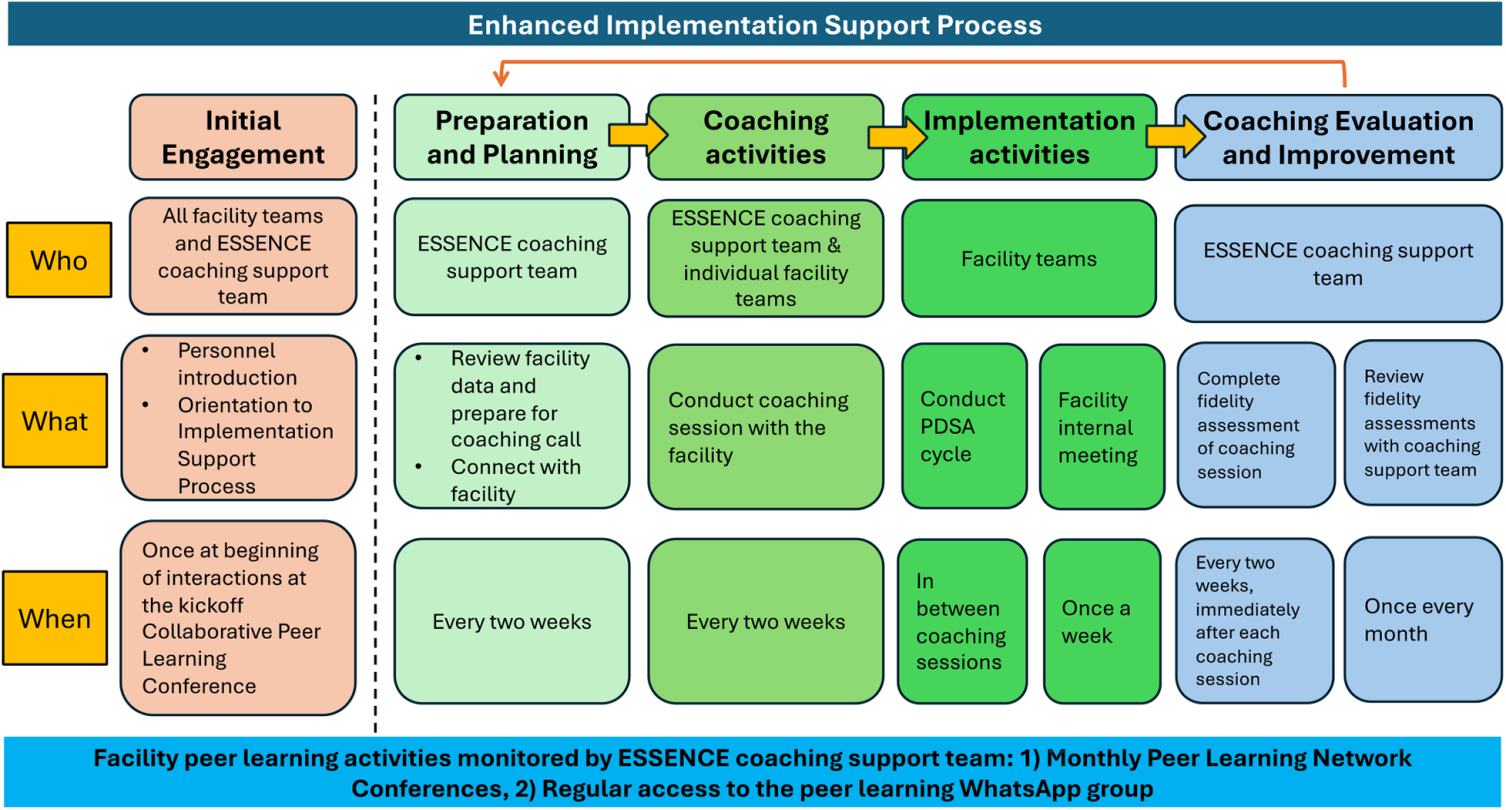
Overview of the enhanced implementation support coaching strategy for intervention arm facilities.

The coaching protocol is guided by the Evidence-Based System for Innovation Support (EBSIS) framework (42, 43), which identifies four critical components for successful implementation of an intervention (i.e., the mhGAP-guided depression care package): (a) adequate training (e.g., training for ANMs/nurses, MOs); (b) appropriate tools at the facility-level to assess and address implementation challenges [e.g., quality improvement enabled using “Plan Do Study Act (PDSA)” cycles]; (c) regular technical assistance and support (e.g., the enhanced implementation support coaching); and (d) quality assurance followed by improvement activities to address implementation gaps. Figure 2 outlines the components of the Enhanced Implementation Support strategy.

The remote technical assistance coaching sessions will be conducted every two weeks with the facility team over phone or videoconferencing platforms (i.e., Zoom). These coaching sessions will involve discussing with the facility team whether they met the performance target they set in the previous session, successes or challenges encountered since the last call, and outlining the plan for the next two weeks. In advance of each call, the coaches will review the facility-level data, which can help guide the coaching session. The calls will involve using the PDSA cycle approach, and offer an opportunity to review performance data, discuss barriers, and identify improvement targets. The coaches will moderate a WhatsApp group to allow follow up and to encourage the facility teams to share progress between coaching calls. There is also a peer learning component where the coaches will host monthly virtual meetings where all the facility teams can exchange challenges, successes, best practices, and lessons learned to collectively problem solve and guide improvement (41, 44).

While the goals of depression care will be based on current NCD program goals (e.g., 100% screening, 100% referral of screened positive cases to the MO), the performance “targets” mentioned under the coaching sessions, will be set during each call. These targets will be revised based on the PDSA cycles and implementation barriers that emerge at the level of each facility. As summarized in Table 2, indicators of successful integration of depression care will be derived from the screening record register, modeled after existing NCD care process indicators. Implementation metrics will be reviewed over the course of the trial, and the Enhanced Implementation Support strategy will intentionally be kept flexible to allow potential modifications, such as including more frequent contact with the facility teams or use of in-person facility visits, to facilitate implementation of the depression care package.

The coaches will complete self-report checklists after each coaching session to monitor fidelity to the coaching protocol. Coaching calls will be audio-recorded, and two members of the research team will listen to a random selection of audio recorded sessions and complete the same checklist to assess fidelity. Rated checklists will be discussed during weekly coach supervision to assess adherence to the coaching protocol and strategies to address implementation barriers. Process data for the coaching sessions will be tracked for each facility, including number of coaching sessions delivered, number and type of facility team members participating in the coaching sessions, number of action plans and PDSA worksheets prepared for each PHC, and time duration of each coaching session.

### Outcome measures

#### Primary implementation outcome

Table 3 lists the study implementation outcomes. For the primary implementation outcome, the proportions of outpatients screened on the PHQ-2 by the ANM/nurse will be compared between study arms. The denominator for the proportion of outpatients screened will be calculated as the total number of adult outpatients for the NCD care program attending the PHCs during the trial period. ANMs/nurses will use a Screening Record Register to document the number of patients screened. The facilities will send the screening data to the data manager on a weekly basis.

**Table 3:** Implementation outcomes.

Measure	Source	Description	Rationale for selection	Hypothesis	Timeline
Primary outcome					
Comparison of Proportion of persons screened on PHQ-2	Screening record register data on primary health center implementation activities, filled regularly by the ANM/nurse	The percentage of adult individuals attending primary health centers who are screened on PHQ-2 by the ANM/nurse.	Screening for depression on PHQ-2 is the gateway for initiating depression care.		Collected/reviewed weekly over the trial duration
Secondary outcomes					
Acceptability: Proportion of patients who refuse to be screened for depression	Screening Record Register data on primary health center implementation activities, filled regularly by the ANM/nurse	The percentage of adult individuals attending primary health centers who refuse to be screened on PHQ-2 by the ANM/nurse will be compared between the trial arms.	Screening for depression on PHQ-2 is the rate-limiting step in the entire patient flow at a primary health center, therefore, assessing the extent of refusal of being screened is important for understanding the acceptability of implementation of depression screening.	The proportion of persons who refuse to be screened on PHQ-2 will be significantly lesser in the intervention arm or in facilities receiving enhanced implementation support, than those receiving routine support (control arm)	Collected/reviewed weekly over the trial duration
Adoption: Proportion of patients initiated on treatment	Screening Record Register data on primary health center implementation activities, filled regularly by the ANM/nurse	The percentage of adult individuals attending primary health centers who are screened positive on PHQ-2 by the ANM/nurse, referred to the MO and initiated on treatment by the MO i.e., provision of psychoeducation and appropriate antidepressant medication and/or referral to the Healthy Activity Program delivered by the ASHA worker, and/or referral to a specialist at the District Mental Health Program or Community Health Centre or private clinic.	Treatment initiation by the medical officer is a comprehensive process indicator as it covers the preceding steps in the patient flow at a primary health center. In addition, this measure reflects the downstream effect of implementation support strategies on adoption of collaborative care package for depression care.	The proportion of screened positive cases initiated on treatment will be significantly greater in the intervention arm or in facilities receiving enhanced implementation support, than in the control arm or in facilities receiving routine support.	Collected/reviewed weekly over the trial duration
Appropriateness, Feasibility, Fidelity (Qualitative measures)	Post-trial exit interviews and/or focus groups with medical officers, ANMs/nurses	Interviews and/or focus groups will discuss experiences of suitability of the integration of mental health services with routine primary care (both arms), practical challenges in achieving the same and extent of adherence to the implementation protocols; Additionally, in the intervention clusters, the discussion will also focus on suitability of the implementation support coaching process as perceived by the facility team, their experiences and challenges of coaching and the extent to which they could adhere to coaching guidance.	Based on Proctor's framework (2010) and the given measures as well as to increase efficiency of resources and time, we have considered a qualitative approach to data collection that we will deploy after completion of trial activities.	Appropriateness, Feasibility and Fidelity of integration of mental health services into routine primary care will be greater in the intervention arm compared to control arm as assessed qualitatively; Within the intervention arm, the appropriateness, feasibility and fidelity of implementation support coaching will be optimum as assessed qualitatively.	End of the study
Cost of Enhanced implementation support: strategy development and delivery costs	Primary health center Facility records; Sangath-Bhopal administrative and finance department for Enhanced Support arm	With regards to development costs, we will include costs for developing Enhanced Implementation Support Strategy (EISS), training the coaches, and piloting/embedding the EISS before formal implementation in the definitive trial. We will capture the costs related to (1) human resources required for developing the EISS, including involved personnel's responsibility, time spent, and salary or payment received, (2) information technology used for development of EISS (e.g., laptops, internet bills, any software etc.), and (3) infrastructure related support such as office supplies, rent, utility etc. Data will be captured via time logs, payrolls etc. by a member of the trial team on a monthly basis. With regards to measuring costs of implementing EISS at seven primary health centres (intervention arm), we will capture spending on (1) health workforce for implementing the EISS (e.g., costs of time spent on coaching facility staff and facility staff's time spent on implementing the EISS-related activities that would enhance their depression screening), (2) technology related costs of coaching (e.g., internet costs, phone bills etc.).	Based on WHO's health system building blocks (WHO, 2010).	Data only collected in the intervention arm	Data will be collected bi-weekly from the beginning of embedding period until the end of the intervention delivery period of the trial.
Exploratory outcomes					
Facility readiness scores and predictors of adoption of collaborative care package for depression	Health Facility Context Assessment (Atlas Initiative Context Assessment Tool, Ariadne Labs), which includes the ‘progress’ survey and the ‘post-implementation’ survey	We have adapted the Atlas toolkit and will use the *Progress* and *Post-Implementation* surveys for trial requirements. The *Progress survey* for frontline staff and facility-leader (medical officer) is a 57-item tool, and *post-Implementation survey* is a 55-item tool. The target audience of these two surveys will include leaders, such as facility-level medical officers in-charge, and frontline healthcare workers involved in delivering the intervention such as ANMs/nurses. Implementation strategy and therefore, look at facility-level factors that are more dynamic than those in the Foundation Survey. The target audience will be similar to that of the Foundation Survey.	In a nutshell, the Progress Survey taps the internal culture of the organisation, which is not expected to change soon after the roll out of the implementation support strategy. The Post-Implementation Survey will assess the need for modifications to the implementation support strategy. The “Progress” Survey will aim to assess the key contextual factors that impact the implementation of a healthcare intervention and should be considered when making decisions about the readiness to implement, the implementation strategy, and possible adaptations to the designed intervention before its delivery. 2. The “Post-Implementation” Survey: The Launch Survey will examine the need to make modifications to the implementation strategy and therefore, look at facility-level factors that are more dynamic than those in the Foundation Survey.	Facilities that show greater readiness scores (regardless of arm allocation) will show greater adoption of the depression care program, resulting in improved implementation outcomes.	The Progress survey will be administered towards the end of embedding as a baseline. The Post-implementation survey will be administered about a month before wrap-up of trial activities.
	The Organizational Readiness for Implementing Change (ORIC) survey	The Organizational Readiness for Implementing Change (ORIC) will be used to assess readiness of the PHCs for implementation of the depression care package.	This 12-item survey is based on Weiner's theory of organizational readiness for change and covers two core constructs—change commitment (5 items) and change efficacy (7 items).	Facilities that show greater readiness scores (regardless of arm allocation) will show greater adoption of the depression care program, resulting in improved implementation outcomes.	This survey will be administered towards the end of embedding as a baseline, and about a month before wrap-up of trial activities.

#### Secondary implementation outcomes

As outlined in Table 3, secondary implementation outcomes will be guided by the heuristic defined by Proctor et al, 2010 (19), and will be assessed using facility-level administrative data and the indicators captured in the screening record register (Table 2), and supplemented with qualitative semi-structured interviews with the facility teams. Implementation metrics will include number of patients who refuse screening, number of patients who screen positive for depression, number of screen positive patients who are referred to the MO and number of patients who are initiated on treatment. Treatment initiation following referral to the MO is an important adoption outcome, and can involve provision of psychoeducation, prescription of antidepressant medication, referral to the brief psychosocial intervention (i.e., HAP) delivered by ASHAs, and/or referral to a specialist at the District Mental Health Program, Community Health Centre or private clinic.

The costs of delivering the Enhanced Implementation Support strategy in the intervention arm PHCs will be collected and categorized into the six building blocks of a health system based on the WHO's health system framework (45). This will include spending on health workforce for participating in the Enhanced Implementation Support strategy activities, such as time required to moderate (for the coaches) and participate (for the ANM/nurse) in the coaching sessions; and information technology, such as internet costs or phone bills for engaging in the coaching sessions. This data will be captured via time logs, payroll, and expense reports. Facility staff salaries will be collected from the National Health Mission reports on salary range for various health personnel in public health facilities. To clarify, the cost analysis will not evaluate the cost-effectiveness or involve a comparison of costs between the study arms because we are not able to collect cost data from the control arm given the irregular or need-based format of routine district team's support to the clinics for delivering depression care (refer, “Routine implementation support”), and because there is no additional implementation support being provided by our team for which the costs could be collected. We will be assessing only the cost of delivery of Enhanced Implementation Support strategy among the intervention arm clinics.

### Qualitative and observational data collection

At the end of the trial, qualitative interviews with facility teams, including ANMs/nurses and MOs, will be used to assess acceptability and feasibility of implementing the mhGAP depression care package across facilities in both arms. These interviews will focus on understanding experiences delivering depression care in both the intervention and control facilities, and to determine whether there may have been differing experiences in facilities receiving the Enhanced Implementation Support relative to control facilities receiving the Routine Implementation Support. The research team will also visit the facilities in-person to further collect observational and qualitative data about facility-level characteristics that may affect implementation of depression care, such as: facility staffing characteristics including workload, leaves, transfers and new appointments; the extent of dedicated physical space and the privacy of this space for conducting depression screening in the clinic; facility staff perceptions about asking questions included in depression screening, views about potential stigma, and about the materials posted in the clinics such as informational posters; MO attendance and their involvement in depression care activities; and screening rates of comparable NCD care programming. These visits will be coordinated in advance with the facility teams.

### Secondary patient outcomes

Table 4 lists patient outcomes that will be assessed at 3 months after enrolment. The proportion of enrolled patients with scores <5 on the nine-item Patient Health Questionnaire (PHQ-9), indicating remission (46), and functional outcomes using the WHO Disability Assessment Schedule (WHODAS 2.0) (47), and symptoms of anxiety using the Generalized Anxiety Disorder scale (GAD-7) (48), will be collected. A 2-week window for collection of follow up assessments from patients will be used to accommodate scheduling and logistics.

**Table 4:** Secondary patient-level outcomes.

Measure	Source	Description	Rationale for selection	Hypothesis	Timeline
Depressive symptoms	Patient Health Questionnaire (PHQ) 9-item assessment tool Kroenke et al. (46)	PHQ-9 score can range from 0 to 27 since each of the 9 items can be scored from 0 (not at all) to 3 (nearly every day). A PHQ-9 total score of 10 or higher is indicative of depressive symptoms. A total score <5 indicates remission.	PHQ-9 can be entirely self-administered, yet, given the rural primary care context, it is feasible and simple to use for the ANM/nurse.	The proportion of patients showing remission linked to the primary health centers receiving enhanced implementation support is significantly lower at 3-month follow up than the proportion of patients showing remission linked to facilities receiving routine support.	3-month follow up
Functional outcomes	WHODAS 2.0 Üstün et al. (47)	WHODAS 2.0 captures the level of functioning in six domains of life including cognition—understanding and communicating; mobility—moving and getting around; self-care—attending to one's hygiene, dressing, eating and staying alone; getting along—interacting with other people; life activities—domestic responsibilities, leisure, work and school; participation—joining in community activities and participating in society.	WHODAS 2.0 is useful for brief assessments of overall functioning in surveys or health-outcome studies, *in situ*ations where time constraints do not allow for the application of the longer version—as may occur during baseline and end-line in this trial.	Patients at end-line in the intervention arm will have significantly lower scores on WHODAS 2.0, or improved functional outcomes, than patients in the control arm.	Baseline and 3-month follow up
Anxiety symptoms	GAD-7 Spitzer et al. (48)	This is a self-report scale, which is a screening tool and severity indicator for GAD. Items are rated on a 4-point Likert-type scale (0 = not at all, 1 = several days, 2 = over half the days, 3 = nearly every day). GAD-7 items describe some of the most salient diagnostic features of GAD (i.e., feeling nervous, anxious, or on the edge and worrying too much about different things).	GAD-7 was created to increase the identification of generalized anxiety disorder cases in the primary care setting. A cut-off score of 10 was identified as the optimal point for sensitivity (89%) and specificity (82%).	Patients at end-line in the intervention arm will have significantly lower scores on the GAD-7 than patients in the control arm.	Baseline and 3-month follow up

### Exploratory outcomes

The Organizational Readiness for Implementing Change (ORIC) (49) will be used to assess readiness of the PHCs for implementing the depression care package. This 12-item survey is based on Weiner's theory of organizational readiness for change and covers two core constructs—change commitment (5 items) and change efficacy (7 items). The Atlas Initiative Toolkit will be used to assess facility-specific implementation factors (50). The toolkit includes two surveys, beginning with the Progress Survey followed by the Post-Implementation Survey. The surveys are based on an organizational readiness heuristic, abbreviated as R = MC2 (51), which defines organizational readiness for an innovation (R) as a function of three components: motivation to implement an innovation (M), the general capacities of an organization (C), and the innovation-specific capacities needed for a particular innovation (C). The surveys were translated and adapted for use in Madhya Pradesh, India, and will be collected from the MOs and ANMs/nurses involved in delivering depression care. The Progress Survey will be collected before trial launch to assess contextual factors that may impact the readiness to implement depression care. This survey captures the internal culture of the facility, which is not expected to change soon after initiating the Enhanced Implementation Support strategy. The Post-Implementation Survey will be collected from the same facility staff about one month before the end of the 12-month active intervention phase to assess the need for modifications to the implementation strategy.

### Sample size estimation

Based on prior outpatient footfall data from participating PHCs, it is estimated that roughly 178 adult outpatients will attend each PHC per month; thus, over a 12-month period of delivery of the Enhanced Implementation Support strategy, there will be approximately 14,994 patients attending facilities in each arm. Assuming about 10% of these patients [based on prior case detection data in the region (14)] will refuse screening or will be excluded at the discretion of the ANM/nurse, the resulting estimates work out to about 13,494 outpatients available for screening in each arm. This sample size will allow us to detect a 15% difference in the proportion of PHQ-2 screenings between arms, assuming that 10% of patients are screened in the Routine Implementation Support Arm, at 80% power, an inter-cluster coefficient of variation of 0.5 (calculated from background facility data on adult outpatient footfall and existing NCD screening rates) and an intra-cluster correlation (ICC) of 0.05 (52).

Proportions (and not numbers of screenings) will be used to account for the between-facility variation in outpatient footfall.

The earlier PRIME study conducted in the same region achieved a 12% depression screening rate in its facility detection survey component (14), and assuming an additional contribution by the various components of enhanced implementation support (i.e., fortnightly coaching calls, WhatsApp support and monthly peer-learning conferences) to further increase the depression screening rate, we will hypothesize a 25% screening rate in the intervention arm, or a 15% between-arm difference in screening rates. We have referred the formula for calculating sample size for parallel cluster randomized controlled trials with fixed number of clusters (*n* = 14 in this study) by Hemming et al. 2020 (52).

### Facility randomization

**Figure 3:**
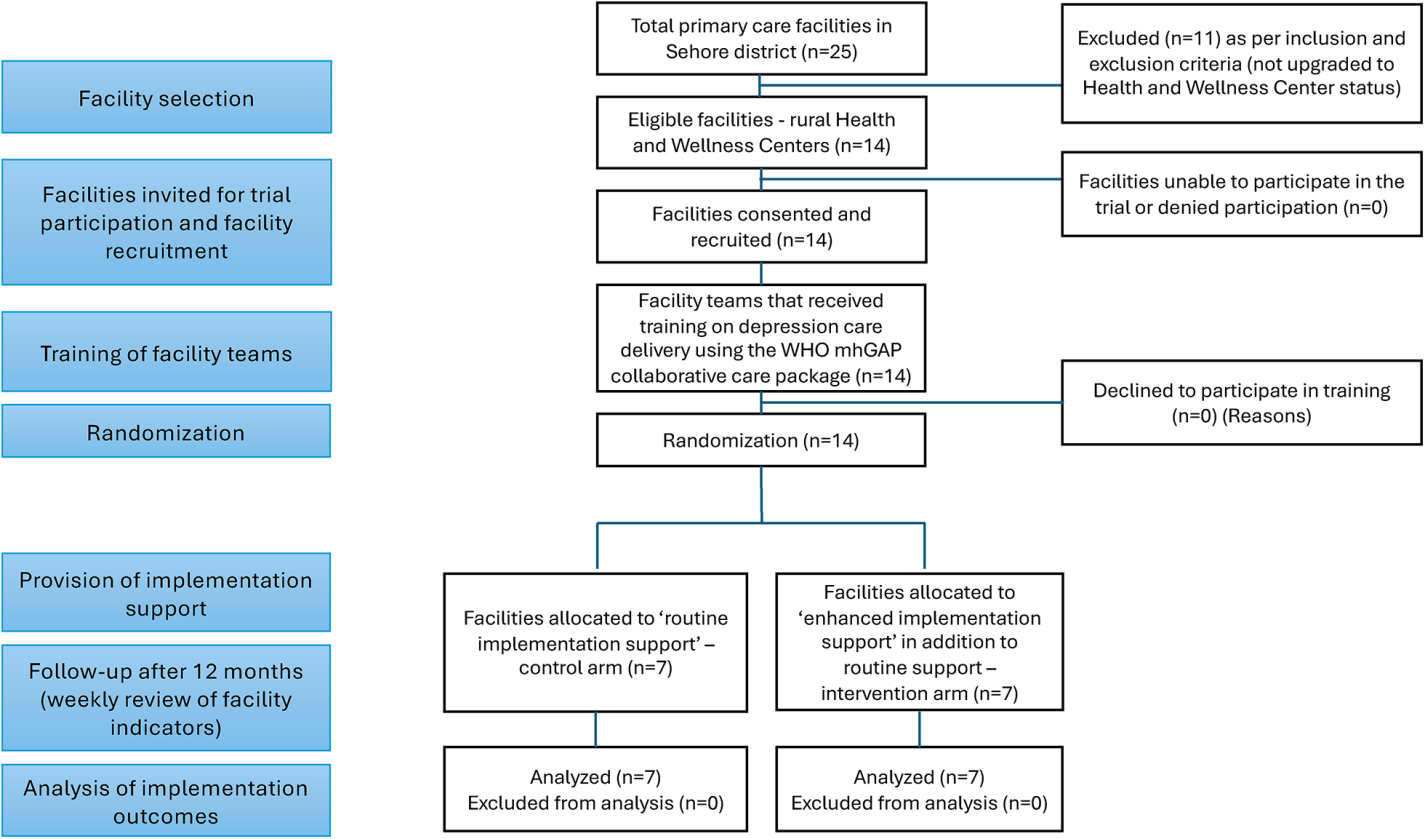
Trial CONSORT diagram.

As outlined in the CONSORT diagram in Figure 3, PHCs will be randomized to the “Enhanced Implementation Support” intervention or “Routine Implementation Support” control condition using 1:1 random allocation. Stata statistical software will be used to prepare the allocation table, to assign and monitor the allocation of PHCs. Prior studies have shown that facility size may affect implementation of mental health services, as large facilities may have more capacity for flexibly utilising resources during implementation of a new evidence-based practice compared to small facilities (53). Therefore, facilities will be stratified by number of monthly outpatient attendees, as this variable can serve as a proxy for facility size, and furthermore, busier clinics may face distinct implementation challenges compared to quieter clinics (e.g., a private location for screening). Drawing from facility characteristics, and based on probability proportional to facility size sampling, the expected contributions (percentages) by each facility per arm per month was calculated. These details will be used to define the strata as either “high” or “low” patient footfall facilities, to ensure balance in facility size between arms.

### Blinding

It will not be possible to blind the PHC staff to arm allocation, and the research team will also not be blind to arm allocation. For patients who enrol, they will not be informed about the allocation of their respective facility, and it is unlikely that they would become aware of arm allocation. Study outcome assessors collecting outcomes from patients, and the statistician who will analyze the final outcome data will be blinded to arm allocation. There will be complete separation between the team members involved in delivering the enhanced implementation support intervention, and those involved in administering the outcome assessments; for example, the implementation support team will be based in Sangath Bhopal office and outcome assessors will work from the district office (rural), closer to the study population.

### Statistical analysis

All analyses of implementation outcomes will be intention-to-treat comparisons between arms. Generalized linear regression models with a log link will be used to compare facility-level implementation outcomes, including the primary outcome of proportion of patients screened on the PHQ-2. ANCOVA analyses will be conducted for comparing secondary implementation outcomes between arms. Time by study arm interactions will be explored using a growth curve model to inspect non-linear trends in the progress of the screening, number of patients who screen positive, referral to MO and treatment initiation. Descriptive statistics will be used for comparing exploratory measures of facility readiness and context assessment between arms. The process indicators from the PDSA coaching sessions will be used for descriptive analysis, to quantitatively assess fidelity to the protocol for Enhanced Implementation Support. Logistic regression models will be used to compare the proportion of patients who remit on the PHQ-9, defined as score <5, between arms at 3-month follow-up. Possible covariates will be entered into the models, including age or gender of the patients, as well as adjusting for cluster effects. Linear regression models will be used to assess differences in the change in disability (WHODAS 2.0) and anxiety (GAD-7) outcomes at 3-month follow up, with baseline values as covariates to adjust for any baseline inter-cluster differences. Stata version 17.0 will be used for all statistical analyses and *p*-values <0.5 will be considered statistically significant.

We will use thematic analysis with a mix of deductive and inductive approaches for coding the transcribed data from qualitative interviews (e.g., post-trial clinic staff interviews) and generating the themes [Braun & Clarke, 2006 (54)]. Thematic analysis will flexibly allow us to include both *a priori* or pre-existing themes from the interview guide, as well as “inductive” themes that will emerge during the analysis. An independent researcher (RS) will develop the initial codes reflecting important areas that we will aim to explore, before reviewing the transcripts and further developing the codes. After independently coding the transcripts, the researcher will refine the codes using inductive/emerging themes, and after consultation with the wider team of academic researchers with expertise in qualitative methods (JN, VP, AB, RR, APB), subsequent iterations will be made to the coding structure i.e., by adding new codes, deleting redundant codes, and integrating the overlapping codes. We will organize consensus meetings to resolve disagreements, such as on the classification of themes.

### Ethical considerations

Institutional Review Boards (IRB) at Harvard Medical School, United States and Sangath, India have approved all study procedures. Additional approval was obtained from the Government of India's Health Ministry Screening Committee, housed at the Indian Council of Medical Research. Written informed consent will be mandatory for enrolling patients in this trial. Participation in the study is completely voluntary and patients can decline participation or withdraw at any time without any consequence to their care at the PHCs. The confidentiality of participants will be protected using unique study ID numbers, and by separating study data from any identifiable data. An independent Data and Safety Monitoring Board (DSMB) will examine accumulating data to assure protection of participants’ safety and data integrity throughout the trial. The research team will submit regular progress reports, including serious adverse event (SAE) reporting, CONSORT flow charts, and baseline characteristics of enrolled participants across study arms to the DSMB. The DSMB will meet before the start of the trial, at the trial mid-point, and at the end of the study to review the statistical analysis plan. The team will maintain a Regulatory Binder containing all required regulatory documents that will be available at any time for study audit. Regulatory files will be checked at the research site for compliance prior to initiation of the trial, throughout the trial, and at trial closure. Study investigators will verify that study procedures are followed and that study staff are trained and able to conduct the protocol appropriately.

From the perspective of collecting mental health data in a rural setting, all participants’ baseline and endpoint assessment and consent records will be kept in password-protected computers/servers. All physical copies of documents will be kept in locked cabinets located inside the Sangath office. All data containing personal identifiers of participants will be delinked by removing all direct identifiers and assignment of a unique ID, combining all main indirect identifiers. We will train and conduct periodic refresher trainings of outcome assessors to manage situations of emotional distress that the participants may experience during the assessments and we will put in place, referral pathways to report distress such as to the medical officer in the primary health centre and the psychiatrist at the district hospital (note that the enhanced implementation support intervention is delivered to the facility teams and not the patients). This will also include management of reported instances of severe adverse events such as reported suicidal ideations/attempts, which the research team will come to know at the patient's three-month follow-up assessment. A study psychiatrist based at Sangath or a tertiary hospital in Bhopal will contact the participant within 24 h of receiving the SAE report, facilitated by the study team if required, to arrange a convenient time and place to complete a detailed assessment either by phone or face-to-face within 7 days, to assess relatedness to trial procedures, and offer any necessary intervention. Finally, we will make sure that outcome assessors receive refresher trainings on administrating informed consent in the rural setting, especially for situations such as reaching out to appropriate legally appointed representatives (family members) in cases of illiterate patients, to avoid sensitive situations where depression screen positivity is potentially disclosed to others in the participant's neighbouring community.

### Trial management

The Senior Management Team, consisting of the principal investigators, site-principal investigator, training program leads, outcome evaluators, and data manager, will provide overall trial leadership and will meet weekly to review trial progress, participant recruitment, data collection, process indicators, and any safety concerns or other issues that may arise.

## Discussion

Given the plans to integrate mental health care under the national rollout of the *Ayushman Bharat* program (29), the knowledge generated from this trial could potentially be advantageous for health systems to guide the implementation of the integration of evidence-based intervention for depression in primary care settings in rural India. The Enhanced Implementation Support strategy relies on widely available human resources and scalable strategies, and draws from existing implementation science studies employing “facilitators”, like the remote implementation support coaches delivering technical assistance to the facilities guided by the model for implementation (49, 55–62).

### Limitations

This study has several limitations that should be considered. First, the intervention requires an external remote coaching team to support the facilities in delivering depression care, however, if scaled up, the health system may have staffing or resource challenges to appoint these cadres. This poses significant limitations to the potential for sustainability of the implementation support strategy that will be tested in this trial. Additionally, the remote coaching approach may face challenges due to poor connectivity or low bandwidth, as well as low digital literacy among facility staff. The insufficient support to primary care personnel under the existing district mental health program to deliver depression care (16, 17) is a case in point. Collaborative care models such as the program being rolled out as part of the ESSENCE project should therefore be complemented by strong community outreach to enable communities to seek mental health care and use the clinic-based care models (to enhance case-detection, treatment and follow-up rates), as also recommended by findings of earlier studies in the region such as PRIME (14). To do so, more participatory approaches to involve community members in developing an outreach model is essential, particularly after accounting for their cultural attitudes towards mental health. This will increase community engagement in the delivery of depression care at the clinic-level, which can potentially reduce the intensity of the required external implementation support or additional human resources appointed by the health system, and contribute to a sustained use of remote implementation support. In such a scenario, the external support maybe required for a lesser frequency than in this trial (e.g., monthly coaching calls instead of fortnightly).

Second, while prior formative work revealed an emerging use of technology by primary care personnel in the rural study context (e.g., WhatsApp for routine data reporting by clinic teams to the district team), we anticipate a number of implementation factors that may challenge the delivery of depression care in the clinics, such as staff transfers and re-appointments requiring fresh training, and substantial workload of various existing programs on the nurses and auxiliary nurse midwives that may reduce their engagement in delivering depression care. We want to highlight that since this is an implementation trial rolled out in a rural primary care setting in collaboration with the state government, we will have several opportunities to document these challenges as they influence the implementation outcomes such as depression screening, case-detection and treatment initiation (data on these will be collected on a periodic basis throughout the trial). However, it is important to note the potential limits to generalizability, given that this study will only be conducted in one district, which may not be representative of other low-resource settings in rural India or in other contexts globally. This further emphasizes the need to document any implementation challenges encountered over the course of this trial as a means to better understand the context and whether these insights also inform implementation of depression care in other settings globally. The use of post-trial exit interviews in facilities of both arms will help address these limits to generalizability by allowing us to examine the appropriateness, fidelity and feasibility of depression care delivery given the aforesaid implementation challenges.

Third, as the trial has a hybrid type-III design (28), there is less emphasis on patient outcomes compared to implementation outcomes and we will have limited scope within the trial design and timelines to collect data on long-term mental health outcomes or patient quality of life (though we are gathering disability data of the participants via WHODAS 2.0). Fourth, the role of ANMs/nurses in determining eligibility and willingness of the screened positive to participate in the trial may introduce a selection bias. We had considered the approach of integrating baseline and endpoint assessments of depression (through PHQ-9) in routine primary care activities at the facility, so that all screened positive patients across clinics of both arms can be evaluated for depression outcomes, which can also enhance between-arm comparability of patients. However, this approach involved challenges such as workloads on nurses, auxiliary nurse midwives and doctors to perform additional depression-related assessments in the clinic. We had also considered the alternative of community-based screening of depression via frontline health workers, such as phase-1 PHQ-2 screening at the village-level (by the ASHA) followed by phase-2 PHQ-9 screening at the clinic-level, but there were similar issues of substantial workload on frontline workers. Therefore, we acknowledge the limitation of a potential selection bias in relation to the assessment of our secondary patient outcomes.

### Trial status

After the embedding phase, the trial launched on October 6th, 2022. The 12-month delivery of the Enhanced Implementation Support was completed on October 5th, 2023. The trial closeout was completed on April 24th, 2024, with primary data analyses to follow.
